# Outcome of Hematopoietic Stem Cell Transplantation in patients with Mendelian Susceptibility to Mycobacterial Diseases

**DOI:** 10.1007/s10875-021-01116-1

**Published:** 2021-08-13

**Authors:** Nesrine Radwan, Zohreh Nademi, Su Han Lum, Terry Flood, Mario Abinun, Stephen Owens, Eleri Williams, Andrew R. Gennery, Sophie Hambleton, Mary A. Slatter

**Affiliations:** 1 Pediatric Allergy and Immunology Unit, Children’s Hospital, Ain Shams University, Cairo, Egypt; 2 Children’s Stem Cell Transplant Unit, Great North Children’s Hospital, Newcastle upon Tyne, UK; 3 Translational and Clinical Research Institute, Newcastle University, Newcastle upon Tyne, UK; 4 Paediatric Immunology, CRB level 4, Block 2, Royal Victoria Infirmary, Queen Victoria Rd, Newcastle upon Tyne, UK

**Keywords:** Mendelian susceptibility to mycobacterial disease, Hematopoietic stem cell transplant, Inborn errors of immunity, Immune reconstitution syndrome

## Abstract

Predisposition to mycobacterial infection is a key presenting feature of several rare inborn errors of intrinsic and innate immunity. Hematopoietic stem cell transplantation (HSCT) can be curative for such conditions, but published reports are few. We present a retrospective survey of the outcome of 11 affected patients (7 males, 4 females) who underwent HSCT between 2007 and 2019. Eight patients had disseminated mycobacterial infection prior to transplant. Median age at first transplant was 48 months (9 -192); three patients were successfully re-transplanted due to secondary graft failure. Donors were matched family (1), matched unrelated (3), and mismatched unrelated and haploidentical family (5 each). Stem cell source was peripheral blood (9), bone marrow (4), and cord blood (1). TCRαβ/CD19 + depletion was performed in 6. Conditioning regimens were treosulfan, fludarabine (4), with additional thiotepa (in 8), and fludarabine, melphalan (2); all had serotherapy with alemtuzumab (8) or anti T-lymphocyte globulin (6). Median hospital stay was 113 days (36–330). Three patients developed acute grade I-II skin and one grade IV skin graft versus host disease. Four patients had immune-reconstitution syndrome. Two reactivated cytomegalovirus (CMV), 1 Epstein-Barr virus, and 3 adenovirus post HSCT. Nine are alive, 1 died early post-transplant from CMV, and the other was a late death from pneumococcal sepsis. Patients with active mycobacterial infection at HSCT continued anti-mycobacterial therapy for almost 12 months. In conclusion, HSCT is a successful treatment for patients with mycobacterial susceptibility even with disseminated mycobacterial infection and in the absence of an HLA matched donor.

## Introduction

Mendelian susceptibility to mycobacterial disease (MSMD) is a group of rare inborn errors of immunity (IEI) characterized by selective susceptibility to mycobacteria including BCG-derived *Mycobacterium bovis* and environmental mycobacteria [1, 2]. The main underlying pathogenic mechanism is impaired production of or responses to interferon gamma (IFN-γ) [3, 4]. In addition, mycobacterial susceptibility is a prominent feature of several other non-SCID, non-CGD disorders that also confer vulnerability to other pathogens and are thus classified separately by the IUIS [5, 6]. In common with classical MSMD, these disorders generally impair intrinsic and innate immunity.

Mycobacterial infection complicating non-SCID IEI shows a wide range of clinical manifestations, from localized to disseminated, acute to chronic infections, plus immature or mature granulomas [7–9]. Typically, age of onset is in childhood, but there are reported cases in adults [4]. Owing to BCG vaccination at birth in many parts of the world, some affected newborn infants may present as a consequence of this vaccination [5]. Some patients develop non-typhoidal *Salmonella* infection[8–10], and a significant proportion experience mucocutaneous candidiasis[4]. In some disorders, viral infections, in particular due to herpesviruses, have been reported [8, 9]. Standard hematological and immunological screening results for IEI are often normal [1], making diagnosis challenging. The overall prognosis for MSMD depends on its specific molecular basis but is often poor [11]. Although patients with some genetic mutations benefit from recombinant IFN-γ, treatment of mycobacterial infection may not be curative without correction of the underlying condition as is the case with absent IFNGR where hematopoietic stem cell transplantation (HSCT) is the only treatment [11].

There are few reported data on patients transplanted for MSMD or related disorders conferring mycobacterial susceptibility. We report outcome of patients transplanted in our center, excluding those with CGD who have recently been reported [12, 13]. Patients 9 and 11 have previously been published [14, 15].

Clinical and laboratory data were retrieved from patients’ medical files and laboratory records. Written informed consent was obtained from the parents or legal guardians as per institutional practice.

The donor hierarchy was (i) matched family donor, (ii) matched unrelated donor, followed by a single antigen mismatched unrelated or haploidentical donor. High-resolution HLA typing was performed for class I and II alleles. Six products underwent TCRαβ/CD19 + depletion using the Clinimacs (Miltenyi Biotech Ltd, Surrey, UK) systems [16].

Prior to transplant, all patients were screened for viruses in blood, stool, and respiratory samples including a bronchoalveolar lavage. Routine surveillance for cytomegalovirus (CMV), adenovirus, Epstein Barr virus (EBV), and human herpes virus type 6 (HHV6) in blood was performed weekly. All patients received prophylaxis against fungi, *Pneumocystis jiroveci* (PCP), and viral reactivation and received immunoglobulin replacement until normal IgM was demonstrated. Donor chimerism was measured by labeling peripheral blood with anti-CD3, -CD19, or -CD15 microbeads. Cell lines were separated using an autoMACS® automated bench-top magnetic cell sorter (Miltenyi Biotec Ltd, Surrey, UK). Separated cells were assayed using variable number of tandem repeats.

## Results and Discussion

Between 2007 and 2019, we transplanted 8 children with a history of infection with atypical mycobacteria or disseminated BCG due to 6 different genetic diseases (deficiency of IRF8 (AR), NEMO (*IKBKG*), GATA2, STAT1 (AR), IFNGR2 (AR), gain of function in NFKBIA (AD)). A further 3 patients underwent HSCT for the same genetic disorders in the absence of preceding mycobacterial infection and were included for comparison (IRF8 (AR/AD), GATA2).

Patient characteristics (*n* = 11, 7 female, 4 male) are shown in Table 1. Eight had proven infection, either due to BCG vaccination (4/8) or atypical mycobacteria (4/8). Five (45%) patients presented with failure to thrive, 6 (55%) with lymphadenopathy and or hepatosplenomegaly, and 3 (27%) each had neurodevelopmental delay, eczema, and dental abnormalities.

**Table 1:** Clinical phenotype

Patients	Mutated gene (mode of inheritance)	Age at presentation in months	Sex	Clinical picture	Infections Pre-HSCT	Treatment Pre-HSCT
P1	*IRF8* (AR)	3	F	FTT, neurodevelopmental delay, intracranial calcification with ventriculomegaly, hepatosplenomegaly	Disseminated BCG	Antimycobacterial
P2	*IRF8* (AR)	2	M	Eczema, neurodevelopmental delay, paronychia	Recurrent chest infection, pyelonephritis	None
P3	*IRF8* (AD)	8	M	Small tonsils, hepatosplenomegaly, lymphadenopathy, warts, barrel shaped chest, clubbing	Recurrent chest infections, bronchiectasis, warts	None
P4	*IFNGR2* (AR)	9	M	FTT, neurodevelopmental delay, mycobacterium abscess	*Mycobacterium abscessus*, MRSA in stool	Antimycobacterial
P5	*IFNGR2* (AR)	5	F	Hepatosplenomegaly, lymphadenopathy	Disseminated BCG, cryptosporidium in stool, enterovirus, norovirus, CMV, HHV6, adenovirus, influenza B	Antimycobacterial
P6	*IKBKG* (X-L)	6	M	FTT, eczema, hair loss, ichthyosis, spiky teeth, lymphadenopathy	PCP, norovirus, rotavirus, *Mycobacterium intracellulare* in BAL	Antimycobacterial
P7	*IKBKG* (X-L)	9	M	Hypodontia, ectodermal dysplasia, xeroderma pigmentosa, eczema	Pneumococcal meningitis, disseminated *Mycobacterium avium* infection	Antimycobacterial IFNg
P8	*GATA2* (AD)	2	M		Parainfluenza, moraxella infection	None
P9	*GATA2* (AD, de novo)	168	M	Poor wound healing, vasculitic skin rash, clubbing, oral candidiasis	Recurrent upper respiratory tract infections, disseminated BCG	Antimycobacterial Steroids IFNg
P10	*STAT1* (AR)	1.5	F	FTT, hepatosplenomegaly, lymphadenopathy, skin nodules, jaundice	disseminated BCG, RSV bronchiolitis	Antimycobacterial Steroids Anakinra IFNg
P11	*NFKBIA* (AD, de novo)	1.7	F	FTT, diarrhea, fever, skin rash, hepatomegaly, tooth abnormalities	Salmonella enteritis + osteomyelitis candida esophagitis Disseminated *Mycobacterium malmoense* Sapo- and norovirus	Antimycobacterial

*BAL* bronchoalveolar lavage, *BCG* Bacillus Calmette–Guérin, *CMV* cytomegalovirus, *F* female, *FTT* failure to thrive, *HHV6* Human herpes simplex virus, *HSCT* Hematopoietic stem cell transplantation, *IFNg* interferon gamma, *M* male, *MRSA* methicillin-resistant *Staphylococcus aureus*, *PCP* pneumocystis jiroveci, *RSV* respiratory syncytial virus, *AD* autosomal dominant, *AR* autosomal recessive, *X-L* X-linked

A detailed description of transplant characteristics is summarized in Table 2.

**Table 2:** Transplant details

Patient	Diagnosis	Age at HSCT (months)	Mycobacterial infection	Donor	Source	Conditioning	CD34 + (× 10^6/kg)	CD3 +	GVHD prophylaxis	Outcome	Last chimerism
**P1**	*IRF8*	9	Yes	MUD	CB	FT Alemtuzumab	0.15	2.4 × 10^7/kg	Ciclosporin, MMF	Alive	100% at 3 years
**P2**	*IRF8*	48 (1^st^ HCT)	No	MUD	PBSC	FT Alemtuzumab	8.1	3.7 × 10^8^/kg	Ciclosporin MMF	Alive	100% at 1 year after 2nd HSCT
		93 (2^nd^- HSCT)	No	New MUD	PBSC	FTT Alemtuzumab	9.3	2.4 × 10^8/kg	Ciclosporin, MMF		
**P3**	*IRF8*	97	No	MMUD (mismatch A)	TCRαβ/CD19 depleted PBSC	FTT, ATG, RTX	4.7	0.72 × 10^8/kg	–	Alive	100% at 1.5 years
**P4**	*IFNGR2*	18	Yes	Maternal haploidentical	TCRαβ/CD19 + depleted PBSC	FTT, ATG, RTX	20	CD3 + TCRαβ + 0.43 × 10^4^/kg		Alive	100% at 6 months after 2nd HSCT
		24 (2^nd^- HSCT)	Yes	Maternal haploidentical	TCRαβ/CD19 + depleted PBSC	FTT, ATG, RTX	12.4	CD3 + TCRαβ + 3.3 × 10^4^/kg	–		
**P5**	*IFNGRa*	34	Yes	Maternal haploidentical	TCRαβ/CD19 + depleted PBSC	FTT, ATG, RTX	12.1	CD3 + TCRαβ + 8.2 × 10^4^/kg	None	Alive	100% at 1 year
**P6**	*IKBKG*	54	Yes	MMUD (single A + C mismatches)	BM	FT Alemtuzumab	3.0	5.8 × 10^6/kg	Ciclosporin, MMF	Dead	100% at 7 years
**P7**	*IKBKG*	78	Yes	MMUD (A mismatch)	PBSC	fludarabine, melphalan, Alemtuzumab	4.4	7.3 × 10^7/kg	Ciclosporin, MMF	Alive	100% at 12 years
**P8**	*GATA2*	43	No	MFD	BM	FTT alemtuzumab	4.3	6.3 × 10^7/kg	Ciclosporin, MMF	Alive	T 93%, B 96% CD15 18% at 18 months
**P9**	*GATA2*	192	Yes	MMUD (C mismatch)	BM	fludarabine, melphalan, Alemtuzumab	1.9	3.2 × 10^7/kg	Ciclosporin, MMF	Alive	100% at 11 years
**P10**	*STAT1*	12	Yes	Paternal haploidentical	TCRαβ/CD19 + depleted PBSC	FTT, ATG, RTX	26.5	CD3 + TCRαβ + 3.97 × 10^4/kg	–	Dead	–
**P11**	*NFKBIA*	51	Yes	MMUD (A mismatch)	BM	FT Alemtuzumab	3.6	4.7 × 10^7/kg	Ciclosporin, MMF	Alive	100% at 30 months after 2nd HSCT
		71 (2^nd^- HSCT)	Yes	Paternal haploidentical	TCRαβ/CD19 + depleted PBSC	FTT, ATG, RTX	15.3	CD3 + TCRαβ + 3.1 × 10^4/kg	-		

*ATG* anti-thymocyte globulin, *BM* bone marrow, *CB* cord blood, *GvHD* graft versus host disease, *FT* fludarabine, treosulfan, *FTT* fludarabine, treosulfan, thiotepa, *HSCT* hematopoietic stem cell transplantation, *MFD* matched family donor, *MMF* mycophenolate mofetil, *MMUD* mismatched unrelated donor, *MUD* matched unrelated donor, *PBSC* peripheral blood stem cells, *RTX* rituximab

Eleven patients received 14 transplants. Median age at first transplant was 48 months (range 9–192). Median time lag between presentation and transplantation was 31 months (range 6–89).

One patient had a graft from an HLA-matched family donor (MFD), 3 from matched unrelated donors (MUD), and 5 each from mismatched unrelated donors (MMUD) and haploidentical parental donors. TCRαβ/CD19 + depletion was performed in all haploidentical and 1 MMUD grafts. Stem cell source was peripheral blood (PBSC) for 9 transplants, bone marrow (BM) for 4, and cord blood (CB) for 1, with median CD34 + cell doses of 3.3 × 10^6^/kg for BM, 8.7 × 10^6^/kg for unmanipulated PBSC, 1.5 × 10^6^/kg for CB, and 13.9 × 10^6^/kg in TCR αβ-depleted grafts.

Conditioning regimen used for unmanipulated grafts was either treosulfan (Treo) and fludarabine (Flu) alone (4/8), or with additional thiotepa (TT) (2/8), or Flu and melphalan (2/8), and alemtuzumab (Alem) was used as serotherapy. TCRαβ/CD19 + depleted graft recipients received Treo/Flu/TT with rituximab and anti-thymocyte globulin according to institutional practice. Post-HSCT graft versus host disease (GVHD) prophylaxis with cyclosporin and mycophenolate mofetil was given to all patients except those receiving TCRαβ/CD19 + -depleted products.

Median days to neutrophil and platelet engraftment were 18 and 17 days, respectively. Three patients were successfully re-transplanted due to secondary graft failure. One patient with IRF8 deficiency who had a MUD PBSC with Treo/Flu/Alem conditioning for the first transplant received the second graft from a different MUD with additional TT. A patient with IFNGR2 deficiency received a second haploidentical TCRαβ/CD19 + -depleted PBSC. The patient with NFKBIA gain of function lost the graft following a MMUD BM with Treo/Flu/Alem conditioning but achieved sustained engraftment from a haploidentical TCRαβ/CD19 + -depleted PBSC. There is limited data on the addition of thiotepa to treosulfan and fludarabine, but it is increasingly being used to try to improve engraftment in reduced toxicity regimens. A report from 3 Israeli centers documented 44 patients, who received treosulfan-based conditioning for non-malignant diseases. A comparison in engraftment rates was made between those who received treosulfan and fludarabine (66.7%), treosulfan and cyclophosphamide (16.7%), and treosulfan, fludarabine, and thiotepa (94.7%). This did not translate into any difference in overall or disease free survival [17]. Nine of 11 patients are alive. One patient with autosomal recessive STAT1 deficiency died early post-transplant due to CMV pneumonitis. Another child with NEMO deficiency succumbed 8 years post-HSCT from pneumococcal sepsis. He had normal immune reconstitution, but post-transplant colitis was on topical oral budesonide and 6 weekly infliximab. He had stopped immunoglobulin replacement and was on pneumococcal prophylaxis and awaiting pneumococcal vaccination.

Eight patients received total parental nutrition for a median number of 25.5 days. Median hospital stay was 113 days (36–330). Three patients had grade I-II skin acute GVHD treated either by topical steroids, tacrolimus and/or systemic steroids. The patient with late death had grade IV acute skin GVHD that necessitated a prolonged course of combined immunosuppressive treatment and extra-corporeal photopheresis. Five patients had post-transplant immune-reconstitution syndrome (IRES) related to mycobacterial infection, manifesting as fever, raised inflammatory markers, malaise, and chronic relapsing lesions of skin, bones, and/or viscera. All 8 patients with a history of mycobacterial infection received long-term (median 12 months) combination antimycobacterial therapy with 2–4 agents. Those with severe IRES also received judicious anti-inflammatory treatment with steroids and cytokine blocking agents in cases that were steroid refractory (anakinra, infliximab). One patient developed late, renal biopsy–proven, thrombotic microangiopathy 7 months post-transplant. Six patients had viral reactivation: 2 CMV, 3 adenoviremia, and 1 EBV.

Long-term complications were assessed in 8 patients with more than 2 years’ follow up. The only late complication was in a patient with GATA2 deficiency who developed a melanocytic melanoma 7 years post-transplant which was surgically removed.

We investigated the impact of mycobacterial infection on outcome, but numbers were small with only 3 patients who did not have mycobacterial infection pre-transplant. There was a trend for a longer hospital stay in those with mycobacterial infection compared to those without (median of 87 and 71.5 days, respectively). Immune reconstitution was assessed at 6 months according to numbers of CD4 + lymphocytes, with no significant delay in CD4 + reconstitution in those with mycobacterial infection compared to those without (median CD4 + counts of 400 cells/ul and 392 cells/ul, respectively). Patients with more than 2 years follow up maintained chimerism between 90 and 100%. Patient 8 has mixed chimerism at 18 months with good immune reconstitution and no signs of disease.

Careful donor selection and preparation of the patient prior to HSCT including treatment of active mycobacterial infection are extremely important [18]. In our series, only 4 patients had fully matched donors available. The 2 patients that died had mismatched donors, but the rest of the cohort are alive and well. The presence of active or disseminated mycobacterial infection did not affect survival, but there was a trend toward more prolonged hospitalization. Roesler et al. reported a multicenter survey in which 2 children with active mycobacterial infection died post-HSCT and recommended optimal control of mycobacterial infection before HSCT and use of a non-T-cell–depleted transplant from an HLA-identical sibling after a fully myeloablative conditioning regimen [19]. Other authors report that achieving disease remission before HSCT affects outcome and immune reconstitution [20, 21]. Rottman et al. recommended use of non-T-cell–depleted PBSC or BM in order to achieve stable donor chimerism [22]. In our series, 6 out of 9 patients with a good outcome had TCRαβ/CD19 + -depleted stem cells from mismatched donors. New methods of T cell depletion for mismatched grafts such as CD3 + TCRαβ/CD19 + depletion show promising results in terms of good engraftment but reduced risk of GVHD in IEI. In the absence of a suitable mismatched family donor which is usually easier and faster to organize, a mismatched unrelated donor can be used with success [16, 23].

In conclusion, most of the literature to date concerning HSCT for these disorders consists of case reports and advises transplant only if active infection is controlled and there is a fully matched donor. Our series suggests that improvement in conditioning regimens, graft manipulation, and prolonged anti-microbial treatment have made HSCT a successful option for patients with mycobacterial susceptibility including those with disseminated mycobacterial infection and without a fully HLA-matched donor in centers of expertise where these options are available.

## Data Availability

Clinical data files are stored at the Great North Children’s hospital and may be shared according to institutional guidelines.

## Abbreviations

AD: Autosomal dominant
Alem: Alemtuzumab
AR: Autosomal recessive
ATG: Anti-thymocyte globulin
BCG: Bacille Calmette-Guérin
BM: Bone marrow
CB: Cord blood
CMV: Cytomegalovirus
CYBB: Cytochrome b-245, beta
EBV: Epstein Barr virus
Flu: Fludarabine
GATA2: GATA-binding factor 2
GVHD: Graft versus host disease
HLA: Human leukocyte antigen
HSCT: Hematopoietic stem cell transplantation
HHV6: Human herpes virus type 6
IEI: Inborn errors of immunity
IFN-γ: Interferon-gamma
IFNGR: Interferon gamma receptor
IRF8: Interferon regulatory factor 8
MFD: Matched family donor
MMF: Mycophenolate mofetil
MUD: Matched unrelated donor
MMUD: Mismatched unrelated donor
MSMD: Mendelian susceptibility to mycobacterial disease
NEMO: Nuclear factor Kappa B essential modulator
NFKBIA: NF-kappa-B inhibitor alpha
PBSC: Peripheral blood stem cell
STAT1: Signal transducer and activator of transcription 1
Treo: Treosulfan

## Code Availability

This is not applicable.
